# Electric Field‐Controlled Synthesis and Characterisation of Single Metal–Organic‐Framework (MOF) Nanoparticles

**DOI:** 10.1002/anie.202007146

**Published:** 2020-08-20

**Authors:** Peter D. Morris, Ian J. McPherson, Martin A. Edwards, Reza J. Kashtiban, Richard I. Walton, Patrick R. Unwin

**Affiliations:** ^1^ Department of Chemistry University of Warwick Gibbet Hill Road Coventry CV4 7AL UK; ^2^ Department of Chemistry University of Utah Salt Lake City UT 84112 USA; ^3^ Department of Physics University of Warwick Gibbet Hill Road Coventry CV4 7AL UK

**Keywords:** electrochemistry, metal–organic frameworks (MOFs), nanoparticles, nanopipettes, resistive pulse sensing

## Abstract

Achieving control over the size distribution of metal–organic‐framework (MOF) nanoparticles is key to biomedical applications and seeding techniques. Electrochemical control over the nanoparticle synthesis of the MOF, HKUST‐1, is achieved using a nanopipette injection method to locally mix Cu^2+^ salt precursor and benzene‐1,3,5‐tricarboxylate (BTC^3−^) ligand reagents, to form MOF nanocrystals, and collect and characterise them on a TEM grid. In situ analysis of the size and translocation frequency of HKUST‐1 nanoparticles is demonstrated, using the nanopipette to detect resistive pulses as nanoparticles form. Complementary modelling of mass transport in the electric field, enables particle size to be estimated and explains the feasibility of particular reaction conditions, including inhibitory effects of excess BTC^3−^. These new methods should be applicable to a variety of MOFs, and scaling up synthesis possible via arrays of nanoscale reaction centres, for example using nanopore membranes.

## Graphical abstract

Electrochemical control over the synthesis of nanoparticles of the metal–organic framework (MOF) HKUST‐1 is achieved using a nanopipette injection method to locally mix metal salt precursors and ligand reagents. The MOF nanocrystals are collected and characterised on a TEM grid. Single nanoparticle crystallisation is controlled and monitored in real time.

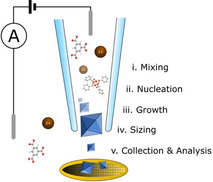

Metal–organic frameworks (MOFs) are a class of hybrid crystalline materials which consist of metal cations or oxo‐clusters, coordinated with organic linkers. MOFs are an area of interest in both research and industry due to their structural stability, ease of functionalisation and high internal surface areas.^1^ MOFs have a variety of applications,^2^ such as catalysis,^3^ analyte sensing,^4^ drug delivery^5^ and as membranes designed for gas separation, storage and purification.^6^, ^7^, ^8^ Achieving a degree of control over the size distribution of nanoparticles of MOFs is key to several applications,^9^ while the ability to generate MOF nanoparticle “seeds” is useful for making MOF films and membranes,^10^, ^11^, ^12^, ^13^ and for mediating the synthesis of pure‐phase MOFs.^14^ However, the rational design of synthetic strategies to obtain small particles is hampered by a lack of mechanistic information about the very earliest stages of nucleation and growth.

The prototypical carboxylate MOF, HKUST‐1, considered herein, consists of Cu^2+^ linked by tridentate benzene‐1,3,5‐tricarboxylate (BTC^3−^) ligands, themselves forming {Cu_2_(BTC)_4_} paddlewheel secondary building units.^15^ It has become the backbone of research into the formation mechanisms of MOFs,^16^, ^17^, ^18^ due to its thermal stability, high porosity towards various gases and relative ease of synthesis.^19^ The simplest synthesis of HKUST‐1 involves stirring an aqueous solution of H_3_BTC with a copper salt precursor.^20^ Such syntheses are highly sensitive to the copper precursor, with acetate (AcO^−^) salts leading to rapid precipitation to form hierarchically porous structures, in contrast to the well‐defined crystallites generated from Cu(NO_3_)_2_ or CuCl_2_.^20^, ^21^ However, aqueous syntheses offer very poor control over growth, making isolation of small seeds with a well‐defined size distribution difficult. More controlled crystallisation is achieved when dimethyl sulfoxide (DMSO) is used as the solvent,^22^ with mixtures of Cu^2+^ and H_3_BTC in DMSO remaining stable towards crystallisation until an antisolvent, such as methanol (MeOH), is added. Preparation of tightly controlled HKUST‐1 (nano)particles for seeding applications remains challenging, however, with slow nucleation continuing into the growth period at typical 1:1 Cu^2+^/H_3_BTC ratios.^18^, ^23^ A powerful strategy for size control of MOFs may be to confine the reagents spatially, by carrying out reactions in small pores in membranes or pipettes.

Nanopipettes, glass capillaries pulled to a taper, provide a facile way to separate two solutions via a nanoscale pore. When an electric field is applied across a solution in a nanopipette, myriad opportunities open up for nanoscale chemical delivery,^24^, ^25^, ^26^, ^27^, ^28^, ^29^ and particle size analysis, via resistive pulse sensing (the Coulter counter principle),^30^, ^31^, ^32^, ^33^, ^34^ with nanopore sizes comparable to analyte size yielding the greatest sensitivity.^35^, ^36^ While the Coulter counter is usually applied to stable particles, it has recently been used to explore nanoscale precipitation in real time,^37^, ^38^, ^39^, ^40^ as well as control crystal growth to generate nanoparticle seeds.^37^ Here we introduce the use of nanopipettes to control fluid and reagent mixing in MOF (HKUST‐1) synthesis and highlight the resulting benefits for the quality of the crystals produced and the possibility of characterising the reaction in real time. In a first approach, we exert control over the aqueous synthesis by using the nanopipette for local delivery and mixing of reagents to form HKUST‐1 nanocrystals in solution that are collected on a transmission electron microscopy (TEM) grid for microscopy analysis. Second, we use reactive resistive pulse sensing to detect nanoparticles formed in situ, supported by detailed finite element method (FEM) modelling in order to understand the process and estimate particle size in real time. Our studies reveal how an applied electric field can be used to induce MOF crystallisation through precise control of mixing at the liquid‐liquid interface, and show that a detailed understanding of nanoscale mass transport opens up new approaches for the rational synthesis of nanoscale materials.

Initial experiments examined whether nanopipettes could exert control over aqueous synthesis routes of HKUST‐1. A 30 nm diameter nanopipette was chosen to allow sensitivity towards early precipitates in the range of ≈20–30 nm. The nanopipette was filled with aqueous CuX_2_ (X=NO_3_^−^ or AcO^−^) and immersed in a bath of H_3_BTC in methanol (MeOH) containing a TEM grid at the base (Figure 1 A). The effect of applied potential (across Ag/AgCl wire electrodes in the nanopipette and bath, see SI, Section S1), in controlling the flux of Cu^2+^ injected into the bath, and ion migration in the MeOH phase, was investigated using cyclic voltammetry. When X=AcO^−^, zero current was observed at positive or negative tip potentials. This lack of current indicates that a permanent blockage has formed within the nanopipette. Current was still not observed when larger pipette diameters (up to 200 nm) were used. Scanning transmission electron microscopy (STEM) of the nanopipettes of both sizes revealed them to be completely blocked (Figure S1). This is consistent with the occlusion of the nanopipette by rapid formation of a precipitate that prevented current flow. Rapid formation of a hierarchically porous solid when using a Cu(AcO)_2_ precursor was observed previously, and was attributed to the pre‐existence of Cu_2_(AcO)_4_ dimers in solution which facilitate the rapid construction of the paddlewheel secondary building block via ligand exchange.^20^

**Figure 1:**
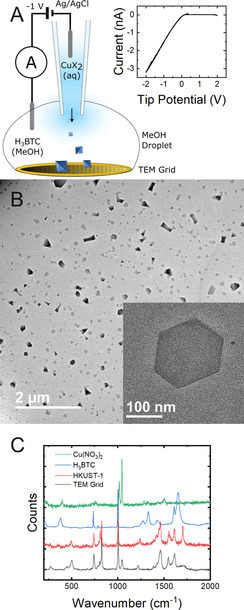
A) Local injection setup for HKUST‐1 crystallisation and deposition onto a TEM grid. Inset: cyclic voltammogram obtained from system. B) TEM image of both truncated octahedral (also shown in inset) and elongated cuboid crystals formed on the grid after experiment, consistent with the morphology of HKUST‐1 and H_3_BTC, respectively. C) Raman spectra of sample of crystals on TEM grid, reference sample of HKUST‐1, and the starting materials H_3_BTC and Cu(NO_3_)_2_.

In contrast, for X=NO_3_^−^, non‐zero current was observed, but only when the nanopipette voltage was negative relative to the bath (Figure 1 A inset). Interestingly, the small extent of H_3_BTC dissociation to H^+^ and H_2_BTC^−^ (mainly) in MeOH was sufficient to pass the currents that flowed (SI, Section S1), but the low ionic strength of this medium compared to the aqueous solution in the nanopipette also resulted in the applied potential field dropping to a considerable extent from the end of the tip into the bath (SI, Figure S14). Thus, at negative tip voltages, the electric field in MeOH impedes the migration of H_2_BTC^−^ towards the tip (and drives any deprotonated forms of H_3_BTC out of the tip), while the very high concentration of Cu^2+^ in the tip causes some diffusion into the bath, but at a slower rate than without the negative tip potential. This ensures the nanopipette itself remains clear of precipitate, but provides a means of controlling the mixing of Cu^2+^ and H_3_BTC just outside the tip (at the nanoscale). In contrast, at positive potentials, there will be a strong flux of H_3_BTC (and deprotonated forms) into the tip, causing the blockage that manifests as no current flow.

Based on these data, steady injection was carried out by positioning the end of the nanopipette a few millimetres above a TEM grid placed inside a MeOH droplet and applying a constant potential of −1 V (a negative potential that sustained mixing without blocking the nanopipette in either solvent system) until the droplet evaporated (≈10 minutes). No impulses were observed in the current, consistent with any crystallisation occurring outside of the nanopipette, as conjectured above. TEM of the grid revealed sub‐micron hexagonal particles, consistent with truncated octahedral HKUST‐1 crystals lying on their (111) face^23^, ^41^, ^42^ as well as elongated particles consistent with the morphology of crystalline H_3_BTC (Figure 1 B).^43^ Truncated octahedral particles with diameters as small as 80 nm were observed with clearly defined facets (Figure S2D). The crystals were not confined to a single region of the TEM grid, because the end of the nanopipette was a few millimetres above the TEM grid, and the methanol solvent was left to evaporate (Section S3, Figure S2). Larger blue crystals were also occasionally observed, visible under a light microscope, and showed a Raman spectrum consistent with a reference sample of HKUST‐1 (Figure 1 C, Table S1). Raman spectra of other regions showed that H_3_BTC residue was also present on the TEM grid (SI, Section 4, Figure S3).^44^, ^45^, ^46^, ^47^ The formation of isolated nanocrystals contrasts to the micron‐sized aggregates often found under these conditions, evidencing the control that can be exercised over crystallisation through the use of nanopipette mixing.^20^

We next turned to the resistive pulse sensing approach to enable in situ characterisation of nanoparticle formation in real time from 100 mm Cu(NO_3_)_2_ and 50 mm H_3_BTC dissolved in a DMSO bath, and 100 mm Cu(NO_3_)_2_ in MeOH in the nanopipette. Batch synthesis measurements have demonstrated that these conditions consistently yield phase pure HKUST‐1, albeit with slower nucleation and growth.^22^ As before, significant current was only observed at negative nanopipette potentials (Figure 2 A, inset). Current transients at −1 V now reveal regions of relatively little variation in current (“quiet” regions, Figure 2 Bi), the stable current being due to the presence of Cu(NO_3_)_2_ in both phases, as well as regions of significant current variation (“active” regions, Figure 2 Bii and Biii). Histogram analysis of the quiet regions show the current to be symmetrically distributed around a single maximum. The active regions also have a similar distribution, but also with either a second distribution on the lower magnitude side (Figure 2 Bii) or an asymmetric tail (Figure 2 Biii). The currents in these features are attributed to resistive impulses occurring as HKUST‐1 particles form in, and translocate through, the nanopipette tip, in accordance with previous “Coulter counter” methodologies.^31^, ^32^, ^33^, ^34^ Note that impulses of similar sizes can have different durations (Figure 2 Biv and Bv). We attribute these variations in impulse duration to different residence times within the nanopipette.^48^ Such variations are often attributed to differences in particle charge, and hence electrophoretic velocity,^30^ however since HKUST‐1 paddlewheel units, and their aggregates, are neutral overall, we attribute these variations to difference in formation position, with formation further away from the end of the nanopipette resulting in longer translocation times.

**Figure 2:**
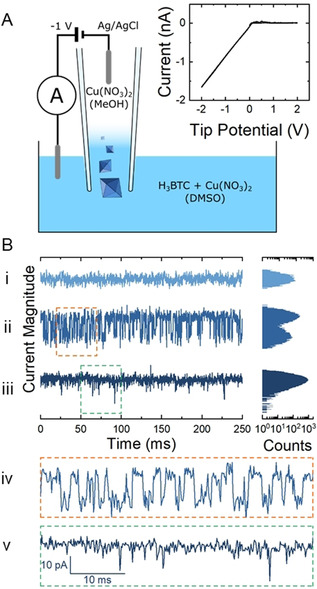
A) Resistive impulse sensing setup. Inset: cyclic voltammogram obtained with 50 mm H_3_BTC + 100 mm Cu(NO_3_)_2_ in the bath and 100 mm Cu(NO_3_)_2_ in the nanopipette. B) Examples of current–time traces at a tip potential of −1 V, for the same conditions as the cyclic voltammogram. Current in quiet regions (i) is symmetric about the baseline, while active regions show additional maxima (ii) or a tail at lower current magnitude (iii). Impulses can last for several ms (iv), or appear as single timepoint fluctuations (v).

Separate experiments were carried out at several different H_3_BTC concentrations in the bath (Table 1) to investigate its influence on the nucleation kinetics and translocation rates. Three nanopipettes were run in parallel for each H_3_BTC concentration, with resistive impulses being observed in at least one nanopipette for each concentration, except for 12.5 mm and 150 mm H_3_BTC concentrations. In the cases of 25 mm and 200 mm H_3_BTC concentrations the baseline current appeared to be significantly lower than the ≈1.3 nA current typically observed (Figures S4 and S9), suggesting an irreversible blockage within the nanopipette. This blockage precluded quantitative interpretation of resistive pules as particle size, indicated by an entry of “n.d.” (not determined) in Table 1. However, in the cases of 50 mm and 100 mm H_3_BTC, regular resistive impulses were observed, along with a baseline current consistent with an unobstructed nanopipette (see simulations below).

**Table 1:** Resistive pulse analysis.^[a]^

[H_3_BTC] [mm]	*f* [s^−1^]	*d* [nm]
12.5	*n.i*.	*n.i*.
25	*n.d*.	*n.d*.
50	41	21±5
100	9.2	20±2
150	*n.i*.	*n.i*.
200	*n.d*.	n.d.

[a] *f*=mean frequency, *d*=modal nanoparticle diameter, n.d.=not determined, n.i.=no impulses observed.

To relate the current impulse magnitude to the size of nanoparticles formed, and to understand the apparent specificity of the reaction towards concentration and voltage, detailed finite element method simulations of the coupled mass transport and electric field in the nanopipette system were performed.^49^ The simulations were based on a realistic nanopipette geometry (Figure S11) and experimental values for the physicochemical properties of the DMSO‐MeOH binary mixture (details in SI, Section S6). The model considered the steady state mixing of DMSO from the bath and MeOH in the tip, the electric field distribution throughout the resulting solution and the transport of Cu^2+^, NO_3_^−^ and H_3_BTC via diffusion, migration and convection. The steady‐state current calculated from the model (1.87 nA) is in reasonable agreement with the current observed in experiments (1.25–1.35 nA). Inspection of the results reveals that most of the voltage drop occurs at the end of the tip, resulting in very high electric field strengths in this region. The focusing of the electric field in this way is a consequence of the tapered nanopipette geometry, with wider cone angles giving rise to greater electric field magnitudes (Figure S15). As a result, any applied voltage significantly perturbs the local ionic environment, with negative tip potentials decreasing the concentration of both Cu^2+^ and NO_3_^−^ below their bulk concentration and positive tip potentials increasing them (Figures 3 A,B, S17). The perturbation results in asymmetric concentration profiles of Cu^2+^ and NO_3_^−^ and, most interestingly, some local net charge separation (Figure S18). This has two important implications: (1) it shows that in these systems applied voltage is able to control the local concentration of Cu^2+^; (2) the local non‐zero charge density generates electroosmotic flow at the tip, which can in theory affect even the uncharged species (MeOH, DMSO and H_3_BTC) as well as the eventual location of nascent HKUST‐1 nuclei (see SI, Section 7.1).

**Figure 3:**
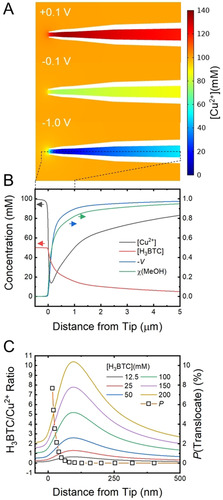
A) Simulated Cu^2+^ concentration in the nanopipette tip at different applied voltages. B) Cu^2+^ and H_3_BTC concentrations, voltage and MeOH mole fraction along the length of the nanopipette when −1 V is applied to the QRCE in the tip. C) H_3_BTC/Cu^2+^ ratio and translocation probabilities, *P*, at −1 V (see text and Supporting Information, Section S5) as a function of distance into the nanopipette from the end.

The H_3_BTC/Cu^2+^ ratio along the symmetry axis, that is, the length of the nanopipette, at an applied potential of −1 V, can be calculated for the different bath H_3_BTC concentrations and the Cu^2+^concentration at the tip (Figure 3 C). Strikingly, the ratio changes significantly in the tip region as compared to either the bulk of the bath or the nanopipette. Significantly, H_3_BTC concentrations higher than 50 mm lead to ratios far in excess of the stoichiometry. It has recently been shown that H_3_BTC has an inhibitory effect on the growth rate of HKUST‐1 at higher concentrations, attributed to the formation of fully coordinated Cu^2+^ ions in preference to the so‐called paddlewheel dimer proposed as the growth unit.^24^ This is entirely consistent with the decrease in the frequency of resistive pulses observed in our experiments. Interestingly, when +0.1 V tip potential is applied in simulations a significant accumulation of Cu^2+^ is observed at the tip end (compare −0.1 V and +0.1 V, Figure 3 A), resulting in a dramatic decrease of the H_3_BTC/Cu^2+^ ratio (Figure S19). However, since excess Cu^2+^ does not show the same inhibitory effect as H_3_BTC, the increase in [Cu^2+^] will instead increase the rate of crystallisation. Thisrationalises the dramatic blocking behaviour (no current flow) observed at positive tip potential in the preliminary cyclic voltammetry (Figure 2 A, inset).

The simulations indicate that at −1 V electroosmotic flow generated at the nanopipette tip is directed from the bath into the nanopipette, further ensuring the mixing occurs inside the nanopipette (Figure S20, see discussion in SI, Section 7.1). As the magnitude of this flow is significant (0.7 mm s^−1^), the question arises as to why translocation impulses are seen, since the flow direction would be expected to strongly affect the ability of nascent HKUST‐1 crystals to diffuse out of the nanopipette and generate the observed resistive impulses. To reconcile the simulated inward flow with the observation of translocation, the probability of a particle diffusing out of the nanopipette against the convection field was estimated using the Fokker‐Planck equation for the drift‐diffusion of probability distributions (details in SI, Section S6.3). The results suggest that although the flow does affect translocation, particles formed close to the nanopipette tip end have a non‐negligible chance of translocating, with the probability rising steeply with closer distance to the tip end (Figure 3 C, S13).

Automated analysis of the current transients enabled extraction of a frequency and particle size distribution for the 50 and 100 mm H_3_BTC experiments, to facilitate a semi‐quantitative comparison (details in SI, Section S5). The current‐time trace was automatically divided into sections of similar background current (determined as the modal current value in that region, Figures S4, S5, S7, S9), such that sections containing resistive impulses yielded asymmetric histograms (Figures S6, S8). A decrease of current magnitude from this baseline defined the threshold for the start of an impulse, while a subsequent increase above a second threshold defined the end of the impulse. Constant absolute currents were used as the start (15 pA) and end (7.5 pA) thresholds for simplicity and were chosen by inspection (SI, Section S5). This enabled the peak current and duration of each impulse to be extracted, although it was not possible to capture all impulses (Figure S10) and, as such, values of *f* and *d* (Table 1) are approximate, although comparable. The peak impulse current was correlated to a particle diameter by comparison with the fractional change in simulated current when a sphere of known diameter was introduced into the nanopipette tip (inset Figures 4, S12, details in SI, Section S6.2).

**Figure 4:**
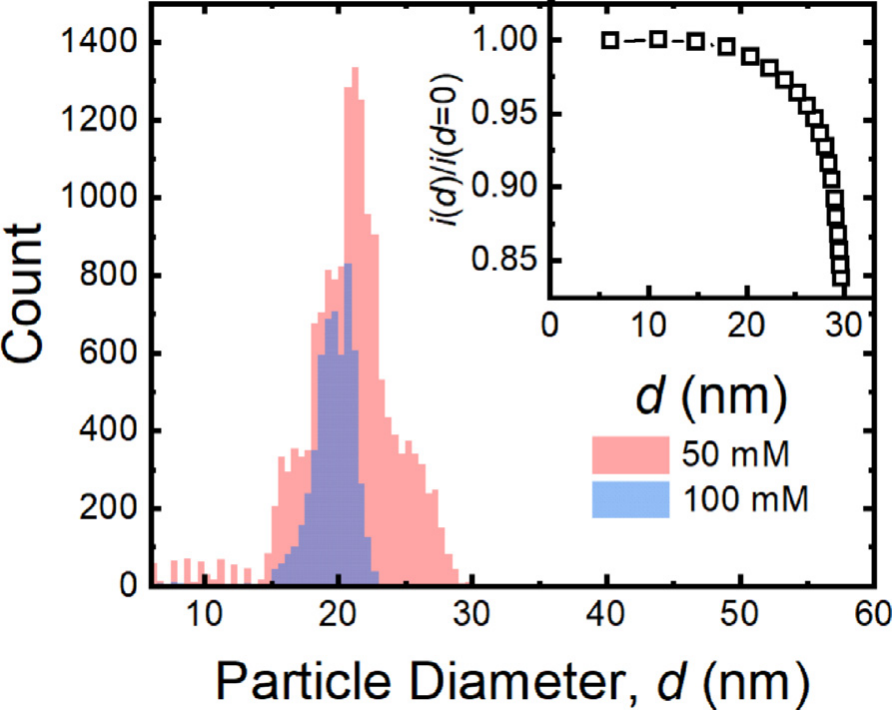
Histogram of particle sizes determined from impulses measured in 50 mm (red) and 100 mm (blue) H_3_BTC + 100 mm Cu(NO_3_)_2_ DMSO solutions. Tip solution: 100 mM Cu(NO_3_)_2_ in MeOH. Inset: calibration curve used to correlate impulse current with particle size.

A decrease in the frequency of particle translocations from 50 mm to 100 mm H_3_BTC is observed, although the apparent particle size remains largely unaffected (Figure 4). This is consistent with fastest nucleation occurring in regions with H_3_BTC/Cu^2+^ ratios closest to stoichiometry (Figure 3 C), in line with previous findings that excess H_3_BTC does not significantly modify eventual particle size, but decreases the growth rate through formation of species other than the paddlewheel growth unit.^50^ The effect of the nanopipette diameter on particle size must also be considered—as the particle grows, it will restrict further mixing of solvent and anti‐solvent. This will begin to limit growth at diameters below the nanopipette diameter and may explain the similarity in observed particle size distributions. Formation of such small nanoparticles could be advantageous if the seeds produced could be collected in some way. This will be the subject of future work.

In summary, we have demonstrated a method for achieving electrochemical control over the synthesis of less‐than 30 nm nanoparticles of HKUST‐1, with nucleation under the confined and defined conditions of a nanopipette. In contrast to conventional approaches to HKUST‐1 synthesis, where a range of crystallite sizes are typically observed due to continuous nucleation during synthesis, we have also been able to use nanopipette injection to form individual nanocrystals that can be collected on a TEM grid and characterised. By changing the reaction conditions, we have also demonstrated the in situ analysis of the size and translocation frequency of nuclei produced with well‐known HKUST‐1 reaction conditions, but translated to a nanopipette format as both a reaction centre and a Coulter counter. We have been able to couple our experimental results with modelling, not only to calculate the size of the nanoparticles, but also to explain why various reaction conditions were successful, or otherwise, in generating particles. Our findings that the frequency of translocations decreases with increasing H_3_BTC concentration is consistent with the inhibitory effect of excess H_3_BTC on the formation of HKUST‐1 through the formation of over‐coordinated species, giving further confidence in our method and analysis, and providing a link between nanoscopic and macroscopic observations. The ability to recover material synthesised using nanopipettes demonstrates this method maybe suitable for generating seeds to use in larger scale synthesis and scale up might be possible via the use of nanopore membranes. The ability to apply different voltages across the nanopipette, as well as to produce nanopipettes of variable size, add further possibilities for detailed control over crystallisation. Given the vast combination of ligands, metal ions, and solvent mixtures, the nanopipette method could be applicable to many MOFs and other hybrid materials, allowing tuning of crystal morphology for practical application.

## Conflict of interest

The authors declare no conflict of interest.

## Supporting information

As a service to our authors and readers, this journal provides supporting information supplied by the authors. Such materials are peer reviewed and may be re‐organized for online delivery, but are not copy‐edited or typeset. Technical support issues arising from supporting information (other than missing files) should be addressed to the authors.

Supplementary
