# Supplementary material for: Electric Field‐Controlled Synthesis and Characterisation of Single Metal–Organic‐Framework (MOF) Nanoparticles
Source: Angew Chem Int Ed Engl. 2020 Aug 20;59(44):19696–701. doi: 10.1002/anie.202007146 (PMC7693291; doi:10.1002/anie.202007146)
Supplement: Supplementary file 1 — Supplementary [file ANIE-59-19696-s001.pdf]

Angewandte Chemie
Eine Zeitschrift der Gesellschaft Deutscher Chemiker

Supporting Information

Electric Field-Controlled Synthesis and Characterisation of Single Metal–Organic-Framework (MOF) Nanoparticles

Peter D. Morris, Ian J. McPherson, Martin A. Edwards, Reza J. Kashtiban, Richard I. Walton, and Patrick R. Unwin**

anie_202007146_sm_miscellaneous_information.pdf

Contents

S1. Experimental Methods S2
S2. STEM Analysis of Nanopipettes S3
S3. TEM Images Following Local Injection Setup S4
S4. Raman Spectroscopy S5
S5. Data Analysis S6
S6. Description of Finite-Element Simulations S13
6.1. Modelling Transport, Flow, and Electric Fields S14
6.2. Evaluating the Resistive Pulse Response S15
6.3. Evaluating the Likelihood of Particle Escape S16
S7. Results of Finite-Element Simulations S18
7.1. Electro-osmotic flow S22
S8. References S24

S1. Experimental Methods

Single-barrelled quartz nanopipettes were used as reaction centres for the precipitation of HKUST-1 and as a Coulter counter for particle analysis. A two-electrode system was employed with an Ag/AgCl wire quasi-reference counter electrode (QRCE) placed into both the nanopipette and the bath, and with the end of the nanopipette being positioned over a TEM grid (Carbon film on Gold 300 mesh, EM Resolutions) in some experiments (see main text). Aqueous solutions were made up using 18.2 MΩ cm water (Purite Select), and all other solvents and chemicals used were purchased from Sigma Aldrich. The nanopipettes were filled with solutions as described in the main text. For all injection and resistive pulse experiments, a bias of –1V was applied to the QRCE in the nanopipette relative to the bath, and the nanopipette was lowered into the droplet and left for a period of ~ 10 minutes. This potential was selected following CV measurements, where the potential was scanned in a cyclic waveform and the current measured, as described in the main text.

For most experiments, nanopipettes were fabricated using quartz glass capillaries with filaments (outer diameter 1.0 mm, inner diameter 0.5 mm, custom manufactured, Friedrich and Dimmock) using a laser puller (P-2000, Sutter Instruments; parameters of: Line 1: Heat 750, Fill 4, Vel 30, Del 150, Pull 80; Line 2: Heat 650, Fil 3, Vel 40, Del 135, Pull 150) to give a tip opening diameter of approximately 30 nm (determined accurately; *vide infra*). Some experiments, where we sought to be able to visualise nanopipette occlusion, employed 200 nm diameter tips (*vide infra*), fabricated from borosilicate glass capillaries with filaments (o.d. 1.2 mm, i.d. 0.69 mm, Harvard Apparatus) using the same laser puller (parameters Line 1: Heat 330, Fil 3, Vel 30, Del 220, Pul -; Line 2: Heat 300, Fil 3, Vel 40, Del 180, Pul 120). The electrometer and current-voltage converter used were home built, while user control of voltage output and data collection was via custom made programs in LabVIEW (2013, National Instruments) through an FPGA card (7852R, National Instruments), with a sampling frequency of 2 µs, averaging 65 samples for a time resolution of 130 µs.

Raman spectroscopy was conducted using a Raman microscope (Horiba LabRam HR Evolution) fitted with a charged couple device (CCD) detector and a 488 nm OPSS laser. A 50× objective was employed for all measurements. Pulled nanopipettes were characterized to determine the inner radius and overall probe geometry by scanning transmission electron microscopy (STEM) using a Zeiss Gemini 500 SEM. TEM analysis was carried out on a doubly corrected ARM200F microscope operating at 200 kV. Annular dark-field STEM (ADF-STEM) images were obtained using a JEOL annular field detector at a probe current of ~23 pA with a convergence semi-angle of ~25 mrad.

When employed as the bath, methanol solutions of H_3BTC were found to be sufficiently conductive even in the absence of supporting electrolyte (as based on the observation that currents, typical of nanopipette experiments, could be measured). Ionisation data for weak acids in non-aqueous solvents are rare, with no data found for H_3BTC dissociation in MeOH. However , it is often found that the shift of the pKa from the non-aqueous to the aqueous value (the medium effect) is approximately constant for groups of closely related acids, with aromatic acids having a medium effect of around 5 in MeOH.[1] Extrapolating to H_3BTC ($pKa_{1,2,3}$ = 3.12, 3.89, 4.70)[2] predicts a pKa_1 in MeOH of 8. A 40 mM solution would therefore be expected to contain 0.6 µM H_2BTC^- and the same amount of cationic species (considering only pKa_1 as an approximation, for simplicity). A coarse simulation of the current, based on the model in section 5 but considering only the ion fluxes and electric field, in a system of pure MeOH with 100 mM $Cu(NO_3)_2$ at the nanopipette boundary and 0.6 µM H^+ and H_2BTC^- at the bath boundary, predicted 0.1 nA at -1 V (see Figure S14, section S7). This is lower than observed experimentally, but of the correct order given the rough nature of the calculation.

S2. STEM Analysis of Nanopipettes

Pipettes used in this study were characterised using STEM, which has been shown to allow accurate determination of their inner and outer diameters.[3] The inner diameter was typically ~30 nm (Figure S1A).

When 30 nm diameter nanopipettes, filled with 100 mM aqueous $Cu(AcO)_2$, were immersed in methanol solutions of H_3BTC, no current was observed throughout voltage sweeps between -1 and +1 V. Absence of current persisted when 200 nm diameter nanopipettes were used in 50 mM H_3BTC in methanol. STEM characterisation of these pipettes shows the formation of globular regions inside the tip consistent with solid precipitate (Figure S1 B,C). Further chemical analysis of this small region inside the nanopipette was not possible.

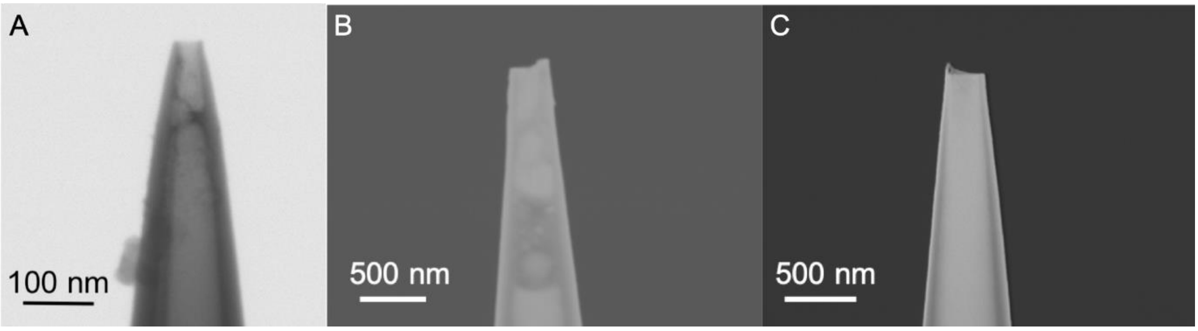

Figure S1: A: Bright field STEM image of 30 nm nanopipette after filling with 100 mM aqueous $Cu(AcO)_2$ and immersion in a solution of 300 mM H_3BTC in methanol. B: Dark field STEM image of 200 nm nanopipette after filling with 100 mM aqueous $Cu(AcO)_2$ and immersion in a 50 mM H_3BTC solution. C: Dark field STEM image of unfilled 200 nm nanopipette, showing no precipitate

S3. TEM Images Following Local Injection Setup

The TEM images below (and in Figure 1B of the main text) were taken following the deposition of HKUST-1 crystals onto the surface of a TEM grid. The end of the nanopipette was ~1 cm above the TEM grid, and the MeOH solvent was left to evaporate, ensuring even coverage of HKUST-1 crystals across the entire grid. Images from various locations on the TEM grid can be seen below, as well as higher resolution images of individual truncated octahedral crystals, showing that even the smallest crystals have a well-defined truncated octahedral shape.

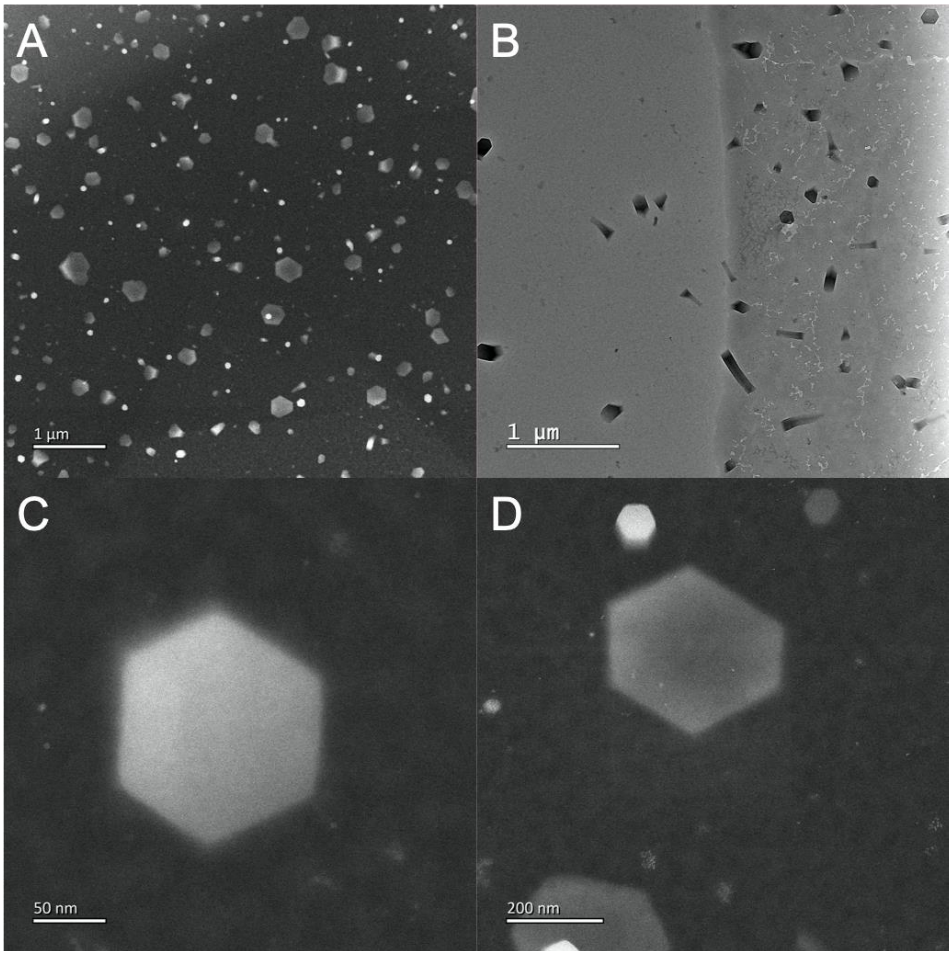

Figure S2: TEM images of various crystals after local injection.

S4. Raman Spectroscopy

Table S1: Assignment of the TEM Grid Raman Spectrum

Wavenumber (cm^{-1})	Peak
274	Cu-O_{water}
452	Cu (II) species
502	Cu (II) species
743	C-H stretch
806	C-H out of plane bending of ring
826	C-H out of plane bending of ring
1006	C=C syn stretch
1046	N-O stretching mode, unreacted $Cu(NO_3)_2$
1221	C-H in plane bending of ring
1421	C-O-O sym stretch
1462	C-O-O asym stretch
1548	C-O-O asym stretch
1614	C=C sym
1712	Carboxylic acid groups

References[4–7]

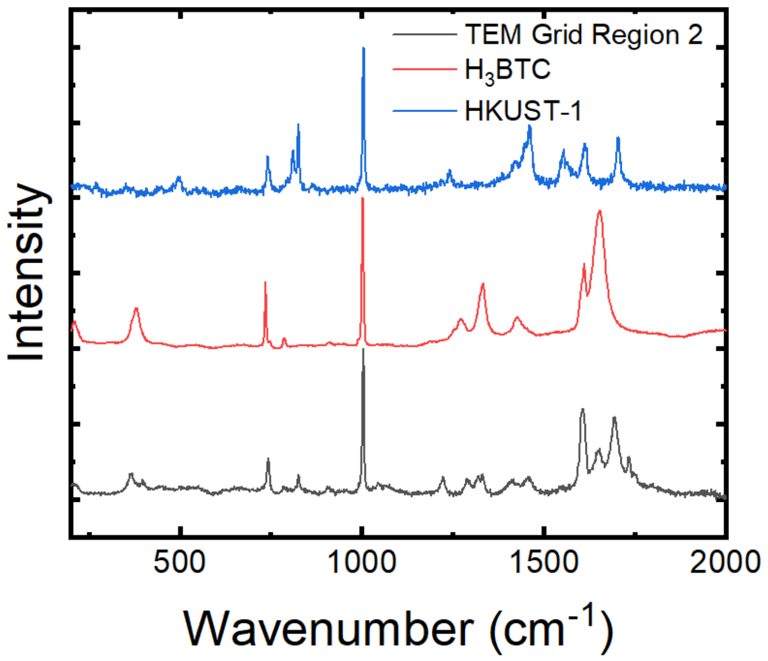

Figure S3: Raman spectra of a different region of the TEM grid to that shown in the main text, showing characteristic peaks of both HKUST-1 and H_3BTC residue.

S5. Data Analysis

Some variations in baseline current and resistive impulse shape during current-time runs were accounted for by automatically separating the data into regions of similar baseline (as determined from the modal current value). The separation algorithm compared a region of data in a given window, splitting the windowed data into two halves and comparing the difference in the modal value and standard deviation, and then splitting the data into four quarters and comparing the difference in modal values between quarters 1 and 2, and 3 and 4. If the differences were less than 5 pA, 20%, and 2 pA, respectively, the region was extracted. If the conditions were not met, the window was shrunk by 25% and the test repeated. If the window was shrunk to a minimum value (0.5 s) without satisfying the conditions, that region was skipped, and the process restarted 0.5 s further along the data. The window was initially set to the entire data set. This enabled a constant local baseline to be determined and subsequent automated analysis of impulses based on a local absolute 'on' threshold (15 pA decrease in current magnitude from the modal value of the section) and 'off' threshold (a subsequent increase in current to 7.5 pA below the modal value of section). The use of absolute (rather than percentage) changes simplified the analysis program and was justified based on the corresponding small percentage change, making it relatively insensitive to changes in the baseline current (for example, a 15 pA threshold for a 1500 pA baseline corresponds to a 1% threshold, while for a 1300 pA baseline it is similar at 1.15%; and these example baseline changes are extreme compared to those in the experiments analysed). Occasionally, the baseline current was far below the value expected, based on simulations of the ion flow, suggesting blockage of the nanopipette. Such data were excluded from the analysis (see examples below). A minimum region size of 0.5 s was used and sections of data failing to meet the criteria were excluded from the analysis (indicated by the absence of an indicated baseline in Figures S2, S3, S5 and S7).

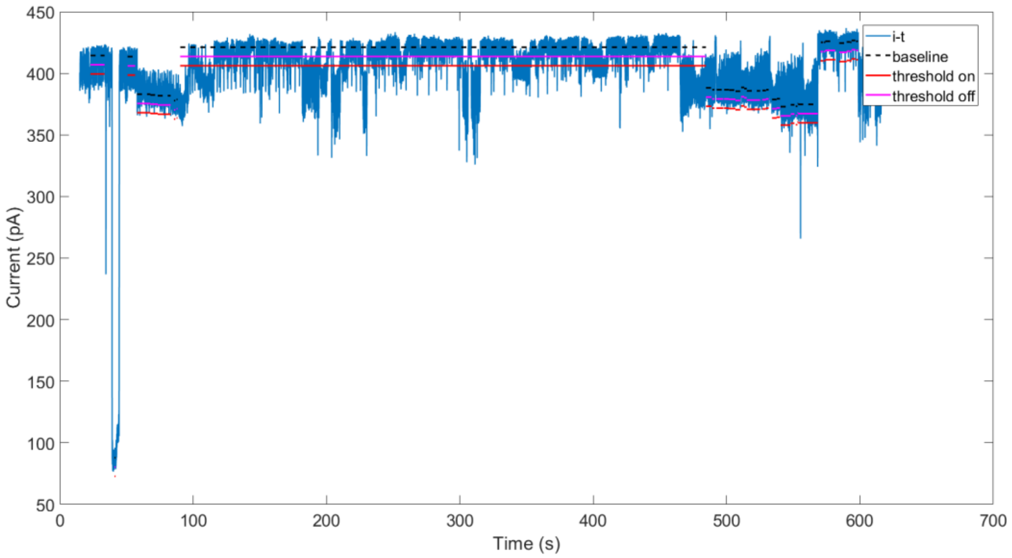

Figure S4: Raw data, and modal baseline and 15/7.5 pA impulse detection thresholds of the extracted regions, measured in a 25 mM H_3BTC + 100 mM $Cu(NO_3)_2$ DMSO bath solution. The lower current magnitude observed in the experiment compared to the value observed in other transients and the simulated current suggests a blockage in the nanopipette, and so the data in the extracted regions were not analysed in this trace.

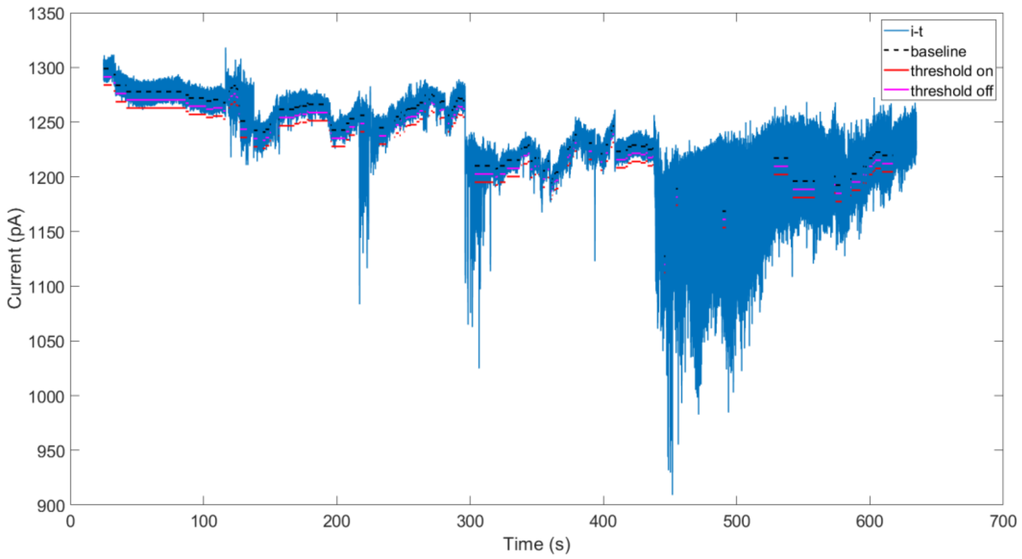

Figure S5: Raw data, and modal baseline and 15/7.5 pA impulse detection thresholds for the extracted regions, measured in a 50 mM H_3BTC + 100 mM $Cu(NO_3)_2$ DMSO bath solution. Examples of the extracted regions are shown below in Figure S6.

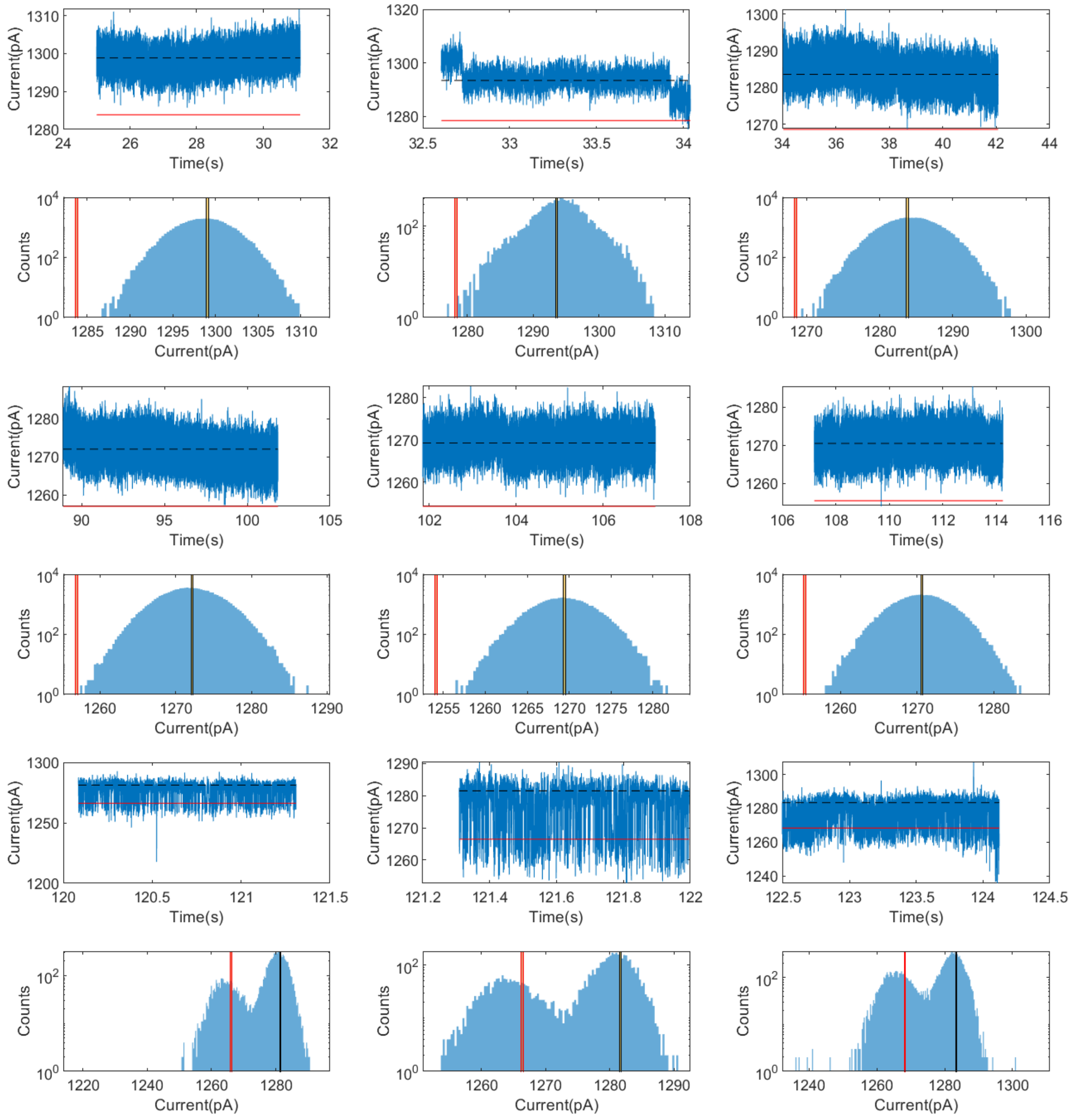

Figure S6: Example analysis on sections of data measured in a 50 mM H_3BTC + 100 mM $Cu(NO_3)_2$ DMSO bath solution (full trace in Figure S5). For each section the raw current-time trace is shown above a histogram of the current. The baseline (black dashed line) is calculated from the modal value of the section and the impulse ‘on’ detection threshold (red line) is set 15 pA below this.

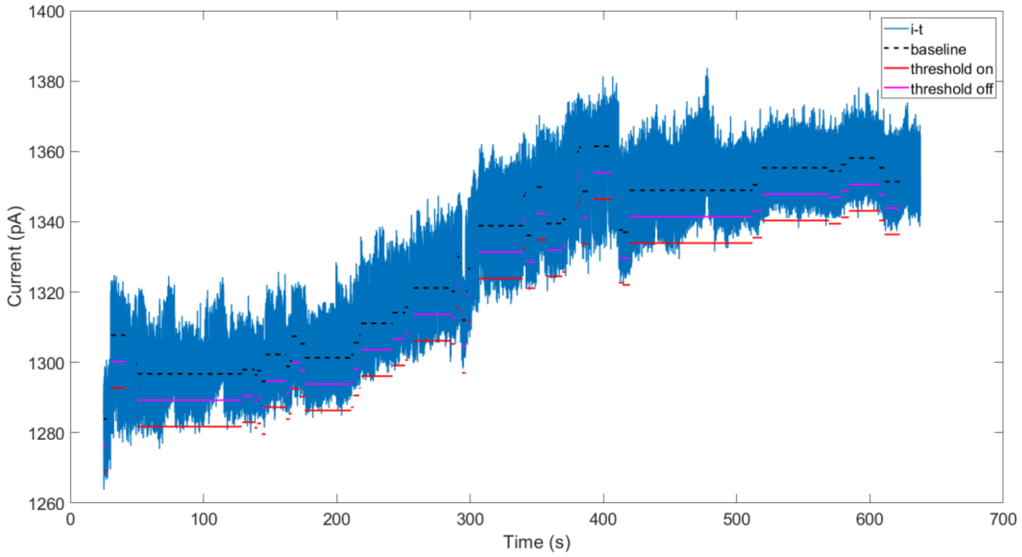

Figure S7: Raw data, and modal baseline and 15/7.5 pA impulse detection thresholds for the extracted regions, measured in a 100 mM H_3BTC + 100 mM $Cu(NO_3)_2$ DMSO bath solution. Examples of extracted regions are shown in Figure S8, below.

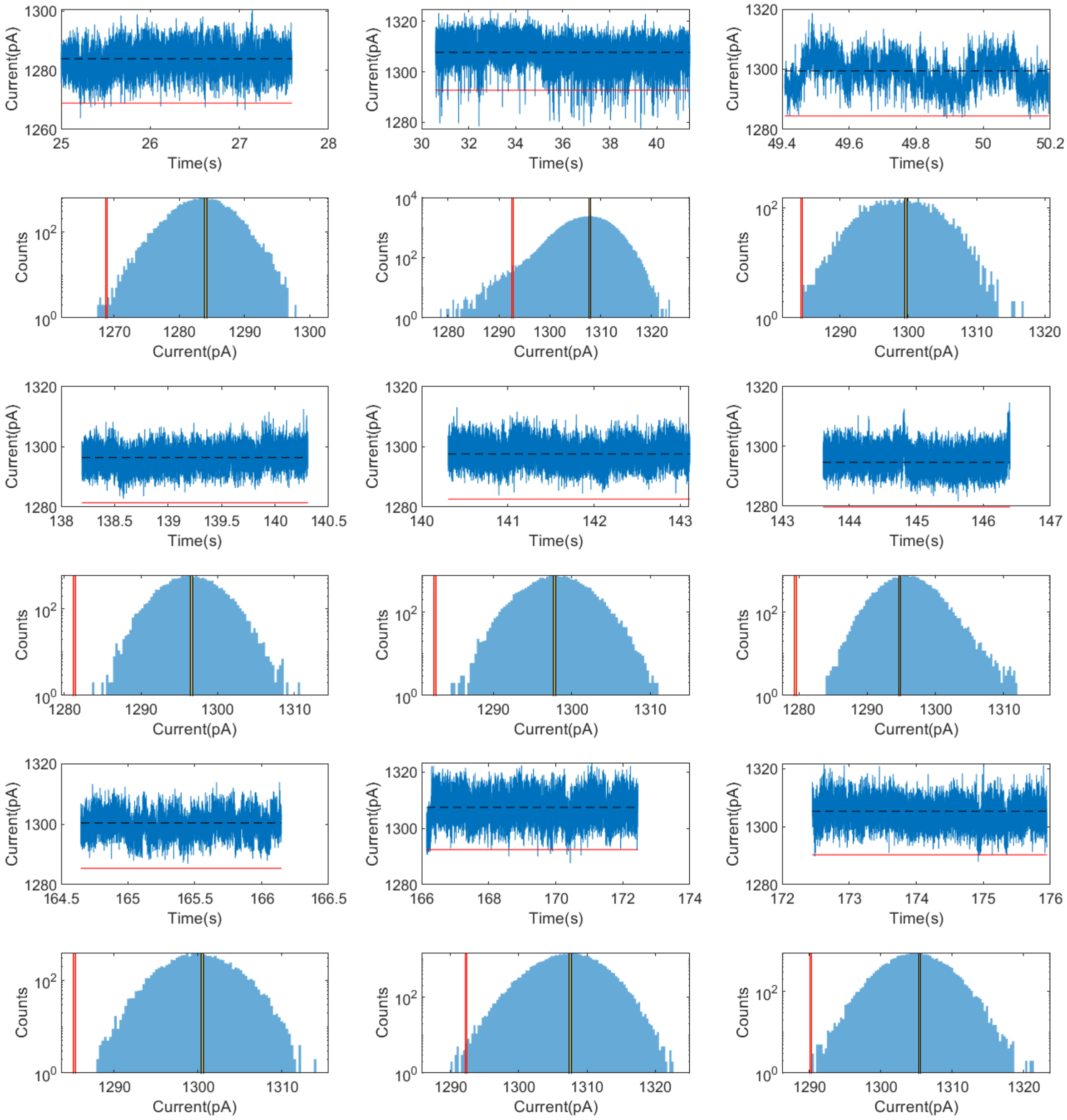

Figure S8: Example of data analysis on sections of data measured in a 100 mM H_3BTC + 100 mM $Cu(NO_3)_2$ DMSO bath solution (full trace in Figure S7). For each section the raw current-time trace is shown above a histogram of the current. The baseline (black dashed line) is calculated from the modal value of the section and the impulse 'on' detection threshold (red line) is set 15 pA below this.

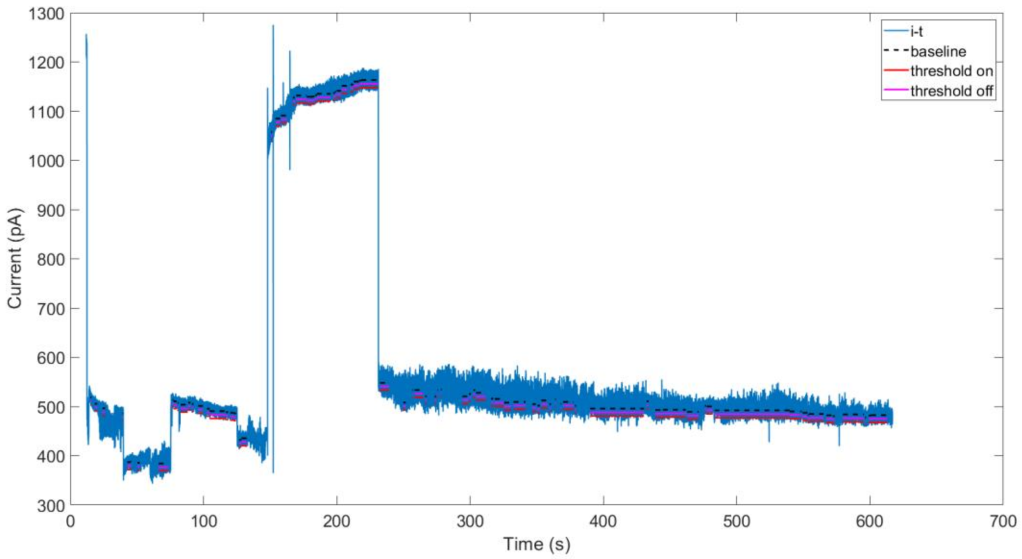

Figure S9: Raw data, and modal baseline and 15/7.5 pA impulse detection thresholds for the extracted regions, measured in a 200 mM H_3BTC + 100 mM $Cu(NO_3)_2$ DMSO bath solution. As in Figure S4, the lower current magnitude observed in the experiment compared to the value observed in other transients and the simulated current suggests a blockage has occurred in the nanopipette, precluding further resistive impulse analysis on the extracted regions.

The distribution of data in a typical region with impulses shows some deviation from a symmetric Normal distribution. This effect can be visualised by mirroring data from the right hand side of the histogram maximum, and shows that the number of data points below the 15 pA threshold exceeds that expected solely from normally distributed baseline noise. The exclusion of these data will lead to an overestimate of the mean impulse current and thus an overestimate of the mean particle size.

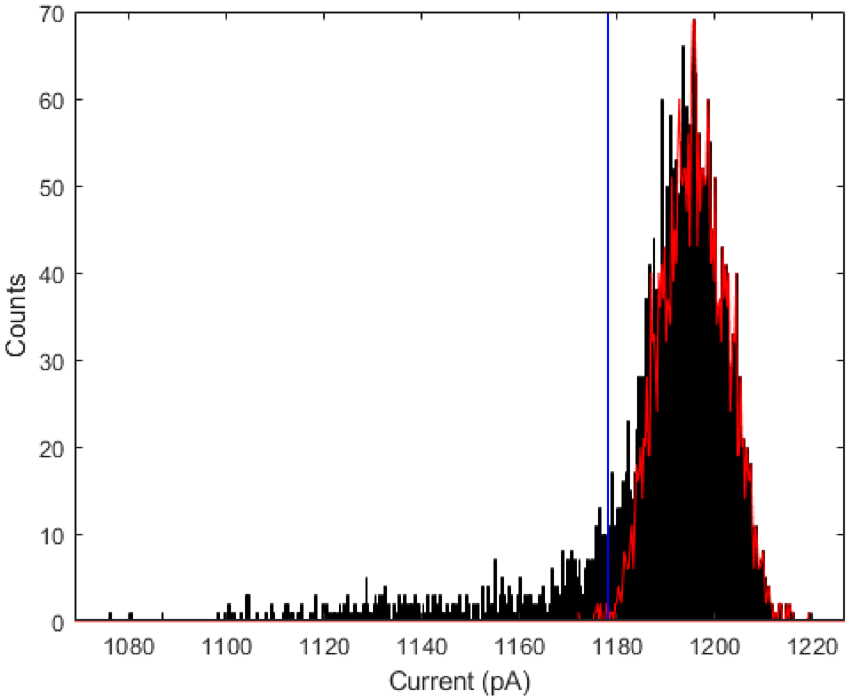

Figure S10: Visualisation of systematic error introduced by the 15 pA threshold. Histogram (black) shows all data points in this region and deviates from the symmetric distribution generated by mirroring data to the right of the histogram maximum (red). The population a currents greater than the threshold (blue vertical line) but above the mirrored data are likely to be impulses which are excluded from the analysis.

S6. Description of Finite-Element Simulations

To understand the relationship between the magnitude of the resistive pulse and the nanoparticle size and to evaluate the plausibility that formed nanoparticles would exit the nanopipette, we performed numerical modelling. The finite-element model developed described the mixing of the solvents, the electric fields, the fluid flows, and the transport of the solutes due to diffusion, electromigration and electro-osmosis. The conditions of the model were taken from the experiments which they described, with the geometry of the pipette taken from STEM images.

The coupled equations describing mass transport, flow and electric field were solved for a steady state using the axisymmetric geometry shown in Figure S11, in which the axis of the pipette is the axis of symmetry. Also shown in the figure are the boundary conditions for the equations and the mesh used.

Below we describe the details of the numerical modelling, which is based on the single solvent model of White and Bund.[8] We start describing the general model, before detailing the modifications used to understand the current response and the likelihood particle ejection form the pipette. In all cases, the model was solved using the commercial finite element package COMSOL Multiphysics v5.5.

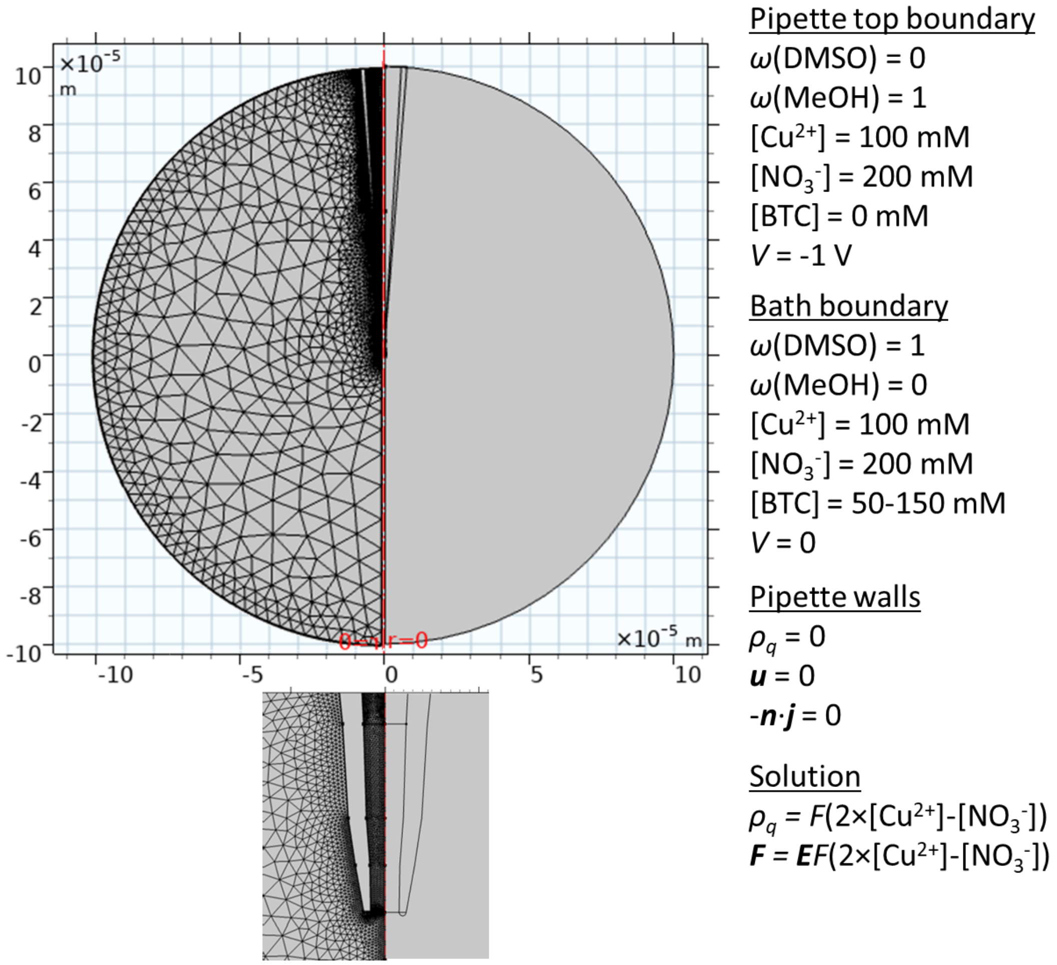

Figure S11: Geometry and boundary conditions used for FEM simulations. Detail of the tip region is shown below. ρ_q is (surface/space) charge density, $\boldsymbol{n}$ the wall normal vector, $\boldsymbol{F}$ the volume force responsible for electro-osmosis and $\boldsymbol{E}$ the electric field. The dashed red line indicates the symmetry axis.

6.1. Modelling Transport, Flow, and Electric Fields

Solvent mixing was calculated using the ‘Transport of Concentrated Species’ module. The mole-fraction dependent density and diffusion coefficients were taken from experimental values, which were interpolated to give a continuous function.[9–14]

Fick’s first law of binary diffusion was assumed for the solvent mixing where the mass flux of solvent *i* relative to the mass averaged velocity, $\boldsymbol{j}_i$, is given by equation S1, with ρ the mixture density, D_i the diffusion coefficient of *i*, ω_i the mass fraction of *i*, M_n the mean molar mass of the mixture (calculated by S2, where M_i is the molar mass of solvent *i*) and $\boldsymbol{j}_{c,I}$ the mixture diffusion correction factor (to maintain the net diffusive mass flux at zero, calculated by equation S3 where x_k is the mole fraction of component *k*).[15] Mass conservation is provided by equation S4, where $\boldsymbol{u}$ is the solution velocity.

$$\boldsymbol{j}_i = -\left(\rho D_i \nabla\omega_i + \rho\omega_i D_i \frac{\nabla M_n}{M_n} - \boldsymbol{j}_{c,i}\right) \quad \text{(S1)}$$

$$M_n = \left(\sum_i \frac{\omega_i}{M_i}\right)^{-1} \quad \text{(S2)}$$

$$\boldsymbol{j}_{c,i} = \rho\omega_i \sum_k \frac{M_i}{M_n} D_k \nabla x_k \quad \text{(S3)}$$

$$\nabla\cdot\boldsymbol{j}_i + \rho(\boldsymbol{u}\cdot\nabla)\omega_i = 0 \quad \text{(S4)}$$

The flux of the solute species *i* = Cu^{2+}, NO_3^- and BTC, $\boldsymbol{j}_i$, were calculated from the Nernst-Planck equation (eq. S5) utilising the ‘Transport of Diluted Species’ module.

$$\boldsymbol{j}_i = -D_i \nabla c_i - \frac{z_i F}{RT} D_i c_i \nabla \mathrm{V} + c_i \mathbf{u} \quad \text{(S5)}$$

The charge on the solute is given by z_i, c_i is the local concentration, and *F*, *R* and *T* the Faraday constant, molar gas constant, and temperature (298 K), respectively. *V* is the electric potential and **u** the solvent velocity. Diffusion coefficients were varied based on the local solvent ratio (Figure S16). These were interpolated from reported experimental conductivities of MeOH/DMSO mixtures, except for H_3BTC, which was estimated based on the Wilke-Chang correlation, using local values for the mean molar mass, density and viscosity at temperature *T*.[9–14]

The electric potential was calculated by solving Poisson’s equation (eq. S6) using the ‘Electrostatics’ module. It contained the space charge associated with local imbalance of Cu^{2+} and NO_3^- and used local values for permittivity, ϵ, based on the local solvent ratio and interpolated literature values,[9–14] where ε_0 is the permittivity of free space.

$$\nabla^2 \mathrm{V} = -\frac{F}{\varepsilon\varepsilon_0}\sum_i z_i c_i \quad \text{(S6)}$$

Given the Reynolds number (R_e ~ 10^{-5} << 1) we used creeping flow (S7) to simulate electro-osmotic flow based on the volume force arising from the space charge.

$$0 = \nabla p + \mu \nabla^2 \boldsymbol{u} - F\nabla V \sum_i z_i c_i \qquad \text{(S7)}$$

In this equation, p is the pressure and μ is the dynamic viscosity.

6.2. Evaluating the Resistive Pulse Response

The effect on the current of a nanoparticle moving through the nanopipette was simulated by introducing a spherical void into the nanopipette tip (Figure S12). For simplicity, we assumed the particle had no surface charge. Simulations were carried out for a range of void diameters. The current was determined by the integration of the ionic fluxes passing through the top boundary of the pipette.

$$\text{Current} = F \int \sum_i z_i \boldsymbol{n} \cdot \boldsymbol{j}_i \qquad \text{(S8)}$$

The current of an open pipette was used to calculate the percentage blockage from the presence of a particle. A calibration plot of normalised current as a function of particle diameter is presented in Figure 4 (inset) in main text.

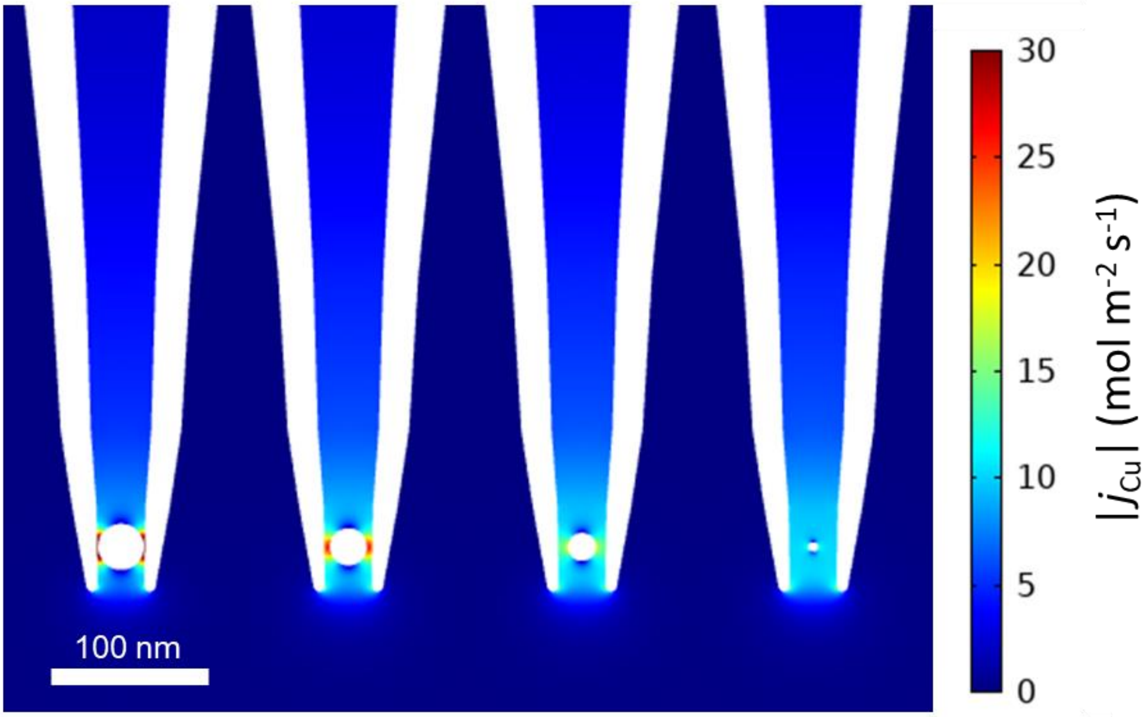

Figure S12: Magnitude of Cu^{2+} flux for nanopipettes partially occluded by nanoparticles of different diameters. Centre of nanoparticle positioned 25 nm from pipette aperture.

Note, while the axisymmetric simulation restricts us the simulating a particle coaxial with the pipette, simulations of resistive pulses in uniform media have shown that the current varies by only ~15% as the lateral position of the particle is varied.[16] This introduces only relatively minor uncertainties in calculating the nanoparticle diameter.

6.3. Evaluating the Likelihood of Particle Escape

To assess the plausibility that a nucleated nanoparticle would exit the nanopipette, we solved the Fokker-Planck equation, which describes the time-varying likelihood (probability density) of finding a particle at a particular position, as a function of time. Such an approach has previously been used to calculate capture efficiencies and distributions of translocation times in nanopore systems.[17,18]

We use the flows calculated as described above. The initial condition is that there is 100% likelihood of finding the particle in the small region representing where the nanoparticle forms. This approach is analogous to releasing a cloud of tracer particles of point size, which possess the diffusion characteristics of the particle (calculated from the Stokes-Einstein equation), and watching where they are transported to.

Integrating the flux of the probability distribution function (PDF) over the aperture of the nanopipette calculates the rate with which the particles leave the pipette. When this is integrated over time, it gives the fraction of particles anticipated to be released.

The probability density as a function of time for two release locations is shown in Figure S13. We can see that initially, the nanoparticle is almost certainly to be found where it is released. However, due to Brownian motion, the region within which one expects to find the particle broadens with increasing time. This distribution skews to the interior of the nanopipette due to the electroosmotic flow. However, the non-zero probability density at the pipette orifice (d = 0 nm) is indicative that a certain fraction of the time a particle formed close to the orifice will escape.

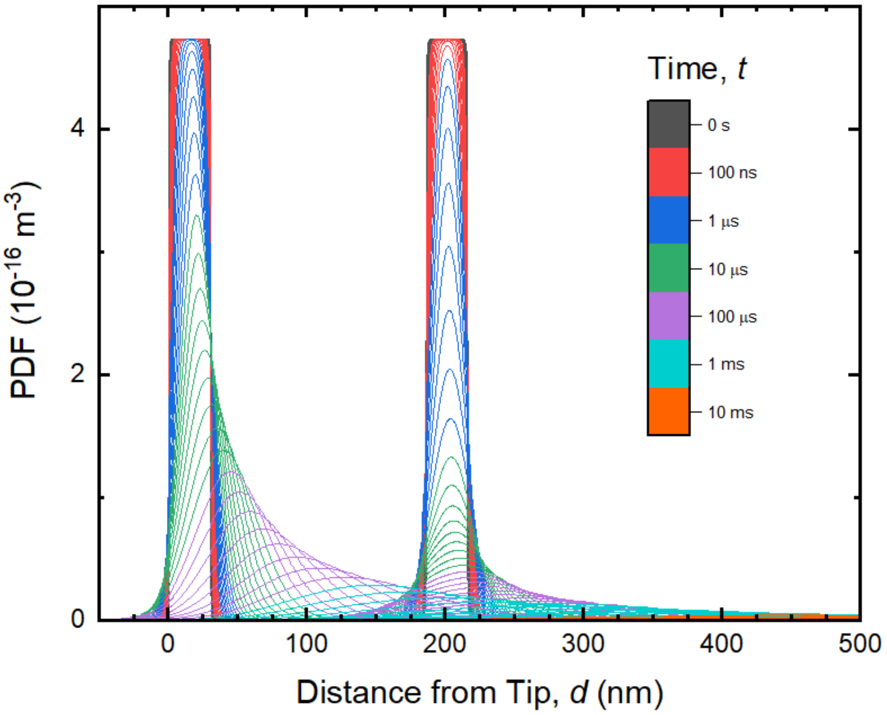

Figure S13: Evolution of probability density function (PDF) taken along the axis of the nanopipette. This function describes the likelihood of finding a particle at different positions within the nanopipette as a function of time. PDF evolution shown for particles released at two positions.

The Fokker-Planck approach approximates a particle as a point, which does not influence the solution of equations S1-S7. It is apparent that a particle of similar size to the pipette diameter will influence the flow and the solute and solvent distribution and thus the results of these simulations should be taken as qualitative in nature. These results show that is likely that some finite fraction of particles formed near the orifice escape the pipette, while particles formed further up the pipette are unlikely to exit the aperture. The simulation of a single translocations of finite sized particle of has previously been demonstrated;[16] however, to perform such a simulations on a large enough number of particles to determine statistics is unfeasible.

S7. Results of Finite-Element Simulations

The model developed above was primarily used for the analysis of the concentration profiles and electric field distribution in the DMSO/MeOH system. However, the same geometry was also used to provide a coarse estimation of the current flow and electric field distribution in the H_2O/MeOH injection experiments, as described in section S1. A solution with a dielectric constant equivalent to methanol (ε = 33) was assumed. The potential at the nanopipette boundary was set to -1 V, concentrations were fixed at 100 mM of M^{2+}, 200 mM X^- ions and 0 mM H^+ and 0 mM H_2BTC^-. The bath potential was set to 0 V and concentrations were fixed at 0 mM of M^{2+}, 0 mM X^- ions and 0.6 µM H^+ and 0.6 µM H_2BTC^-. Diffusion coefficients were all equivalent at 10^{-5} cm^2 s^{-1}. Space charge was included but flow was not considered. The results suggest that the potential drop and resulting electric field will exist at the nanopipette/bath boundary and into the bath. This will limit the ingress of deprotonated H_2BTC^- into the pipette, preventing precipitation inside which would block the nanopipette and prevent further delivery of Cu^{2+} into the bath. The relatively weak field inside the pipette will also hold Cu^{2+} less strongly inside the nanopipette, contributing to its release into the bath. Conversely, a positive applied potential would pull H_2BTC^- towards the nanopipette tip, enhancing the likelihood of precipitation inside the nanopipette orifice, blocking the current.

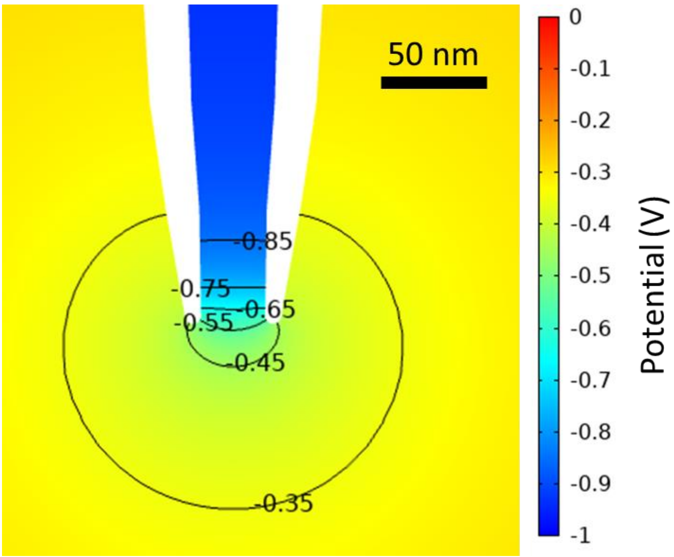

Figure S14 Illustration of the effect of low electrolyte concentrations outside the nanopipette on the potential distribution when -1 V is applied. Contours show equipotentials with values indicated in V.

The following figures were generated from the full model explained in section S6, based on an accurately determined pipette geometry, an applied tip voltage of -1 V, and a BTC concentration of 50 mM unless otherwise stated. 2D plots are constructed from values obtained along the central axis of the nanopipette, where the distance from the tip is positive inside the nanopipette and negative outside.

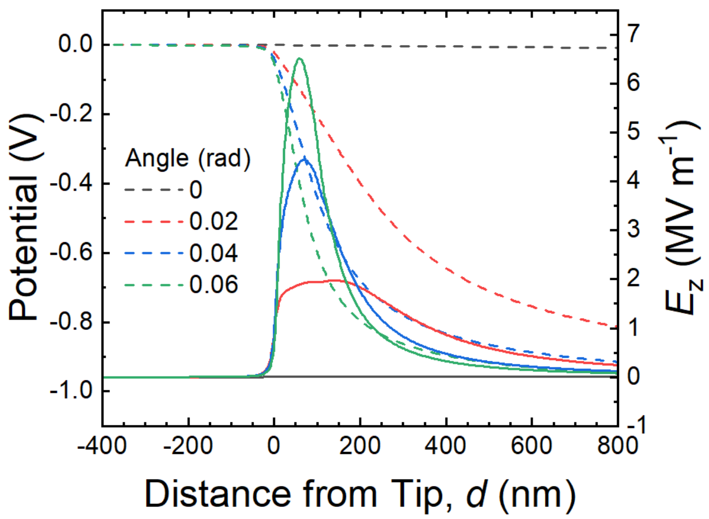

Figure S15: Effect of nanopipette semi-angle on potential (left axis; dashed lines) and *z* component of electric field (E_z, right axis; solid lines) as measured along the central axis of the nanopipette. A simplified geometry (with the diameter only specified at 0 nm, 100 nm and 100 μm along the nanopipette) was considered to enable the angle to be varied (angle indicated in legend, with 0.06 rad the experimental value). In each case -1 V was applied inside the nanopipette vs. 0 V externally.

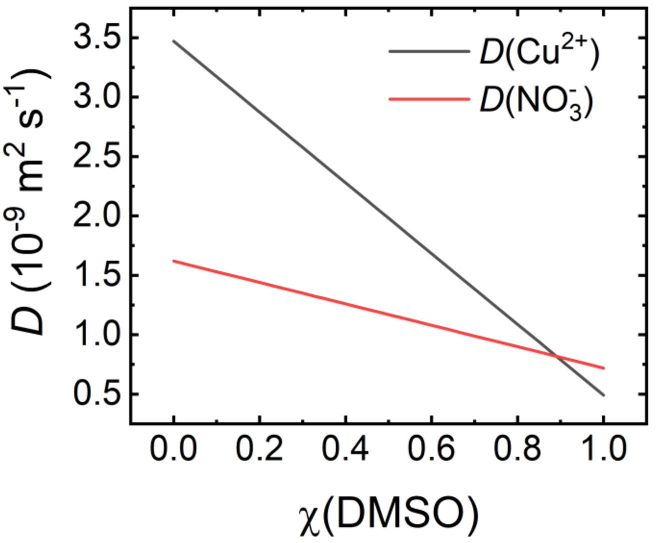

Figure S16 Variation in diffusion coefficients, *D*, for Cu^{2+} and NO_3^-, as a function of DMSO mole fraction, χ. Diffusion coefficients are weighted averages based on experimental conductivities reported in the two pure solvents.[12–14]

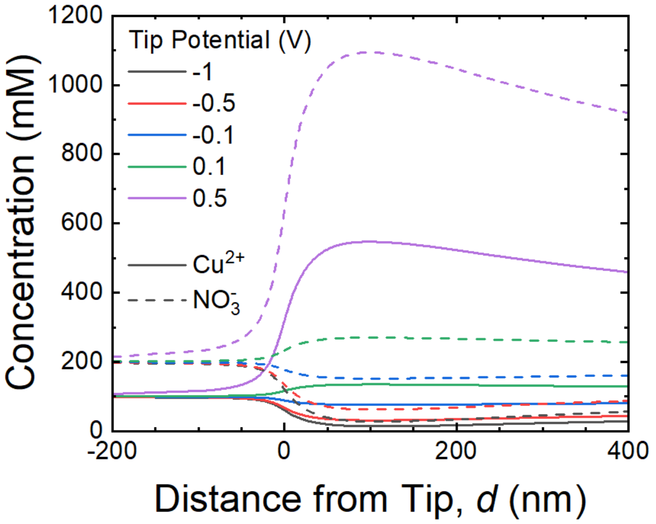

Figure S17: Effect of applied nanopipette voltage on the concentrations of Cu^{2+} and NO_3^- near the tip end, as measured along the central axis of the nanopipette. The full, accurate nanopipette geometry was used. 2D plots of the Cu^{2+} concentration at -1 V, -0.1 V and +0.1 V are shown in the main text (Figure 3A).

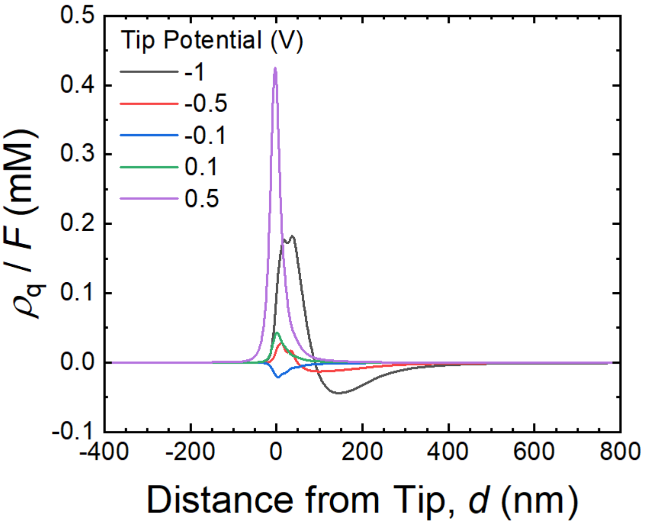

Figure S18: Effect of applied nanopipette voltage on the charge separation of Cu^{2+} and NO_3^- near the tip end, giving rise to space charge density $\rho_q = (2\times[Cu^{2+}]-[NO_3^-])F$.

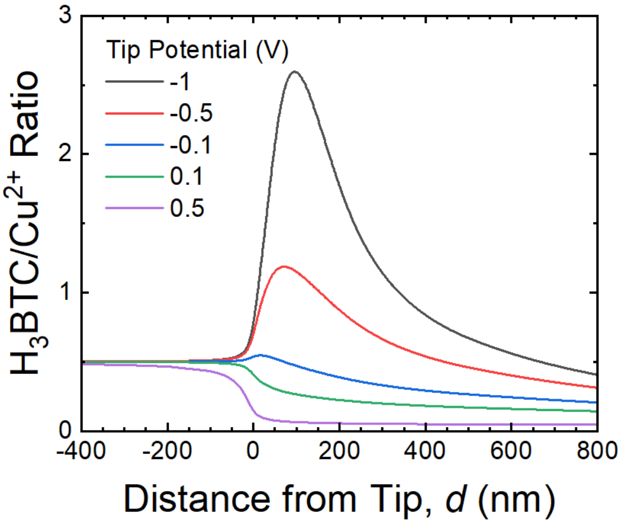

Figure S19: Effect of applied nanopipette voltage on the H_3BTC/Cu^{2+} ratio near the nanopipette tip. H_3BTC concentration was 50 mM. The effect of H_3BTC concentration at constant -1 V applied potential is shown in the main text (Figure 3C).

7.1. Electroosmotic flow

Electroosmotic flow is generated when an electric field exerts a force on a volume of solution possessing a non-zero net charge. It is described quantitatively by the final term on the right hand side of equation S7. If we consider the application of a -1 V bias, as was used in experiments, the electric field (Figure S15, solid lines) is directed into the nanopipette and is largest at approximately 100 nm inside the pipette. The net charge is initially positive inside the aperture of the pipette, before becoming negative further inside the pipette, as can be seen in the black curve in Figure S18. These indicate that the electroosmotic volume force is upward nearer to the tip and downward farther inside. The resulting electro-osmotic flow shown in Figure S20 is directed from the bath as into the nanopipette indicating that the upward force is dominant.

Interestingly, the mechanism by which the space charge forms differs from that reported for glass nanopipettes filled with aqueous electrolytes, in which electroosmotic flow is also observed. In this system, a positive space charge in the double-layer balances that of the deprotonated silanol groups, creating an electroosmotic force. In this work we did not include charge on the nanopipette walls in this model (Figure S11), as surface ionisation behaviour in the solvents used in this work is not well defined. Instead, the charge separation arises from differences in the relative ion transport of the two species (Cu^{2+}/NO^{3-}) in different solvents (Figure S16) leading to an accumulation/depletion in the region just inside the pipette (Fig S18).

In the present system, the relatively low concentration of species and the limited region of high velocity means that the magnitude of the convective flux is much lower than that due to diffusion or migration, such that the concentration profiles remain similar in the absence of simulated electroosmosis.

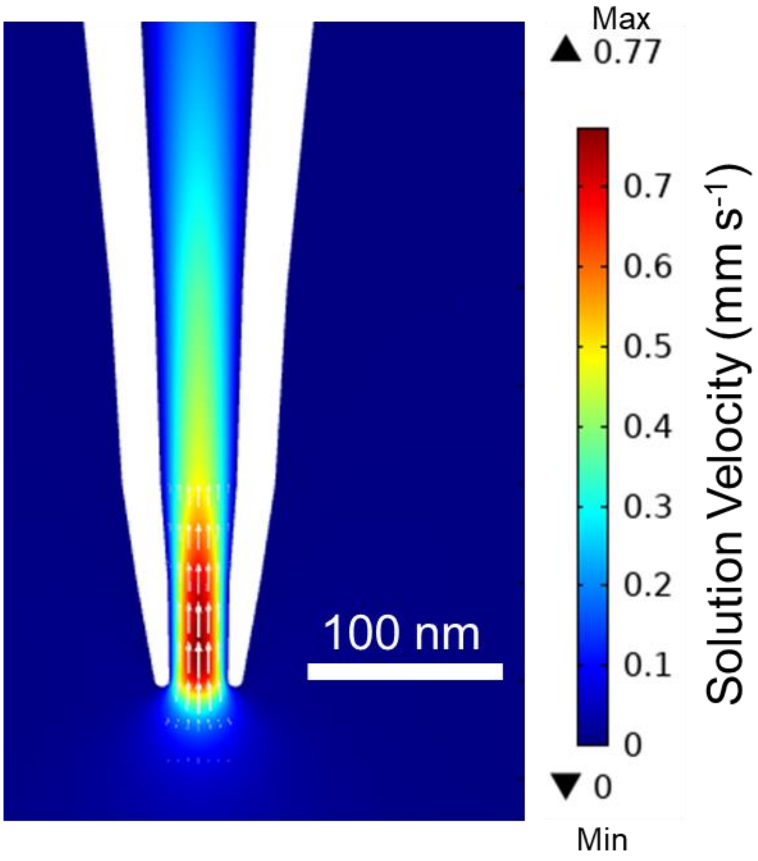

Figure S20: Simulated electroosmotic flow at a nanopipette potential of -1 V. Arrows indicate direction of the flow and have lengths proportional to the velocity magnitude.

S8. References

[1] A. Covington, *Physical Chemistry of Organic Solvent Systems*, Springer Science & Business Media, **2012**.

[2] H. C. Brown, D. H. McDaniel, O. Häfliger, in *Determination of Organic Structures by Physical Methods* (Eds.: E.A. Braude, F.C. Nachod), Academic Press, **1955**, pp. 567–662.

[3] D. Perry, D. Momotenko, R. A. Lazenby, M. Kang, P. R. Unwin, *Analytical Chemistry* **2016**, *88*, 5523–5530.

[4] C. Prestipino, L. Regli, J. G. Vitillo, F. Bonino, A. Damin, C. Lamberti, A. Zecchina, P. L. Solari, K. O. Kongshaug, S. Bordiga, *Chem. Mater.* **2006**, *18*, 1337–1346.

[5] N. R. Dhumal, M. P. Singh, J. A. Anderson, J. Kiefer, H. J. Kim, *J. Phys. Chem. C* **2016**, *120*, 3295–3304.

[6] M. Todaro, A. Alessi, L. Sciortino, S. Agnello, M. Cannas, F. M. Gelardi, G. Buscarino, *Journal of Spectroscopy* **2016**, *2016*, 8074297.

[7] K. E. Ramohlola, G. R. Monana, M. J. Hato, K. D. Modibane, K. M. Molapo, M. Masikini, S. B. Mduli, E. I. Iwuoha, *Composites Part B: Engineering* **2018**, *137*, 129–139.

[8] H. S. White, A. Bund, *Langmuir* **2008**, *24*, 2212–2218.

[9] A. S. Virk, D. J. Codling, T. Stait-Gardner, W. S. Price, *ChemPhysChem* **2015**, *16*, 3814–3823.

[10] C. R. Wilke, P. Chang, *AIChE Journal* **1955**, *1*, 264–270.

[11] P. S. Nikam, M. C. Jadhav, M. Hasan, *J. Chem. Eng. Data* **1996**, *41*, 1028–1031.

[12] B. S. Krumgalz, *J. Chem. Soc., Faraday Trans. 1* **1983**, *79*, 571–587.

[13] H. Doe, T. Kitagawa, K. Sasabe, *J. Phys. Chem.* **1984**, *88*, 3341–3345.

[14] A. K. Mamyrbekova, *Russ J Gen Chem* **2013**, *83*, 1799–1802.

[15] R. B. Bird, W. E. Stewart, E. N. Lightfoot, *Transport Phenomena*, Wiley, New York, **2007**.

[16] Z. Qin, J. Zhe, G.-X. Wang, *Meas. Sci. Technol.* **2011**, *22*, 045804.

[17] B. Lu, D. P. Hoogerheide, Q. Zhao, D. Yu, *Phys. Rev. E* **2012**, *86*, 011921.

[18] J. E. Reiner, A. Balijepalli, J. W. F. Robertson, B. S. Drown, D. L. Burden, J. J. Kasianowicz, *J Chem Phys* **2012**, *137*, DOI 10.1063/1.4766363.

[19] E. J. F. Dickinson, L. Freitag, R. G. Compton, *J. Phys. Chem. B* **2010**, *114*, 187–197.
